# Q fever expertise among human and veterinary health professionals in Germany – A stakeholder analysis of knowledge gaps

**DOI:** 10.1371/journal.pone.0264629

**Published:** 2022-03-03

**Authors:** Fenja Winter, Amely Campe

**Affiliations:** Department of Biometry, Epidemiology and Information Processing, WHO Collaborating Centre for Research and Training for Health in the Human-Animal-Environment Interface, University for Veterinary Medicine Hanover, Hanover, Germany; Guru Angad Dev Veterinary and Animal Sciences University, INDIA

## Abstract

Q fever is a zoonosis caused by *Coxiella burnetii*. In Germany, the common sources of human infections include small ruminants that excrete the pathogen. Q fever in humans can be asymptomatic or nonspecific. However, severe disease progression is also possible, which can lead to death. Q fever in small ruminants is usually asymptomatic, although reproductive disorders may occur. To protect humans from Q fever, it is important that human and veterinary health professionals (practitioners/health authority employees) have comprehensive knowledge of the diagnosis, control and prevention of Q fever, and its zoonotic potential. To ensure and enhance this understanding, this stakeholder analysis assessed Q fever expertise in human and veterinary health professionals in Germany and investigated how these knowledge gaps can best be resolved. For this purpose, an online survey and two focus group discussions were conducted with 836 and 18 participants, respectively. Knowledge gaps are due to a lack of awareness of Q fever, especially among human health practitioners. Moreover, colleagues who have heard about Q fever still lack the necessary cross-species knowledge to successfully diagnose, control and prevent this zoonosis. Additionally, differences exist between stakeholders regarding their work context and the region in which they work. In this study, stakeholders in southwestern Germany had slightly better Q fever knowledge than their colleagues in northeastern Germany. In addition, information sources aimed at resolving knowledge gaps involve direct conversations between the stakeholders, as well as reading materials and seminars. Each of these information sources should focus on interdisciplinary resources to strengthen the cooperation between human and veterinary health professionals and to raise awareness of the strengths of each stakeholder group. These results have already been implemented by the Q-GAPS project, with goals of raising awareness of Q fever and filling knowledge gaps.

## Introduction

Q fever is caused by the small, obligately intracellular, pleomorphic gram-negative bacterium *Coxiella burnetii* that is characterized by high tenacity and virulence [1, 2].

In humans, the inhalation of only a few *C*. *burnetii* organisms may be sufficient in causing infection. Therefore, the inhalation of contaminated dust particles or aerosols is a high risk for humans [2, 3]. The consumption of raw milk or raw milk products, as well as blood transfusions, live cell therapy, obstetrics/childbirth and sexual transmission, are possible (but rare) infection pathways [4, 5]. After infection, acute Q fever is subclinical in nearly 50% of cases. The other 50% of infected people develop unspecific flu-like symptoms, of which high fever and headache are the most common [2, 6]. Further symptoms of acute Q fever include hepatitis, atypical pneumonia, cardiac involvement or neurologic signs [6]. Moreover, chronic fatigue (Q fever fatigue syndrome, or QFS) is possible [7]. Chronic Q fever can occur in the form of endocarditis, hepatitis or neurologic manifestations up to many years after infection [6]. Furthermore, Q fever can lead to abortion, neonatal death, premature birth or intrauterine growth retardation, whereas only scarce data have been available on the risk for pregnancy [2].

The zoonotic potential of *C*. *burnetii* originates from contact between humans and infected animals, such as wild or domestic mammals and ticks, which can shed the pathogen [2, 6]. However, in Germany, small ruminants (sheep and goats) infected with *C*. *burnetii* are the most important reservoir for Q fever in humans [6, 8, 9]. In sheep, infection with *C*. *burnetii* leads to abortion in approximately 5–20% of cases and is otherwise mostly asymptomatic [10, 11]. However, in goats, infection is frequently associated with abortion [12, 13]. Nevertheless, the symptoms of Q fever are not pathognomonic in either species; therefore, a diagnosis is challenging [9, 12, 14]. Infected small ruminants can excrete the pathogen at high concentrations in abortion and birth materials, as well as at lower doses in milk, feces, urine and semen [1, 11, 12, 15, 16]. As a result, contamination of the environment can occur, which means that *C*. *burnetii* can be detected in dust, manure, pastures or wool, and it can also be spread by wind [8]. Conclusively, close and distant contact between humans and small ruminants may cause single infections, as well as large outbreaks, in the human population. Animal owners, their families and employees or veterinarians have frequent close contact with small ruminants and contaminated materials, and thus are at a high risk of being infected with *C*. *burnetii* [2, 3, 9]. Moreover, laboratory staff may become infected via the inhalation of contaminated aerosols in workplaces [4]. Furthermore, general human population experiences close contact with small ruminants when attending conformation shows, open house days, petting zoos, farm vacations, animal-assisted education or therapy. As *C*. *burnetii* can cover long distances via wind (depending on the local geographic and weather conditions), people with distant contact to shedding small ruminants may acquire infection [3, 8, 9, 17]. Although possible, infection via the consumption of contaminated raw milk or raw milk products is rare in Germany. Moreover, the pasteurization of raw milk and food processing inactivates *C*. *burnetii* [16].

The World Organization for Animal Health (OIE) lists Q fever as a multiple species disease. To protect humans from Q fever, the OIE recommends preventive measures, such as protocols for diagnostic testing and vaccinations in small ruminants and cattle [4, 18]. In Europe, the monitoring of Q fever in humans is regulated under Decision No 2119/98/EC and is coordinated by the European Centre for Disease Prevention and Control (ECDC), whereas the monitoring of Q fever in animals is regulated under Directive 2003/99/EG and is coordinated by the European Food Safety Authority (EFSA), the ECDC and EFSA´s Zoonoses Collaborating Centre [19]. In Germany, Q fever is a notifiable disease in both humans and ruminants, with passive monitoring conducted in both groups, although regulations differ [for cases in humans: German Protection against Infection Act, IfSG; for cases in ruminants: German National Animal Health Act, TierGesG, and German Regulation of Notifiable Animal Diseases, TKrMeldpflV; 20–22]. Regional and seasonal accumulations can be identified in human and ruminant populations via data analyses of the reported cases [23–25]. As these systems depend on the awareness of human and veterinary health professionals (among other relevant stakeholders), underreporting has to be presumed, and the true number of (sporadic) cases and (small) outbreaks cannot be reliably estimated [25–28].

Diagnosis, control and prevention of Q fever depend on the expertise of human and veterinary health professionals. Therefore, it is critical to keep these stakeholders at a high level of knowledge. Although nothing is known about the Q fever expertise of human and veterinary health professionals in Germany, we hypothesized that the quality of their expertise influences the number of reported cases. Therefore, because the number of reported cases differs in northeastern and southwestern Germany, we assumed that the expertise of health professionals involved in these regions of Germany would differ. To test these hypotheses, we conducted a stakeholder analysis. This included an online survey on the level of Q fever expertise among human and veterinary health professionals in Germany and two focus groups on how to most effectively fill potential knowledge gaps.

## Methods

### Data collection methods and data management

**Fig 1:**
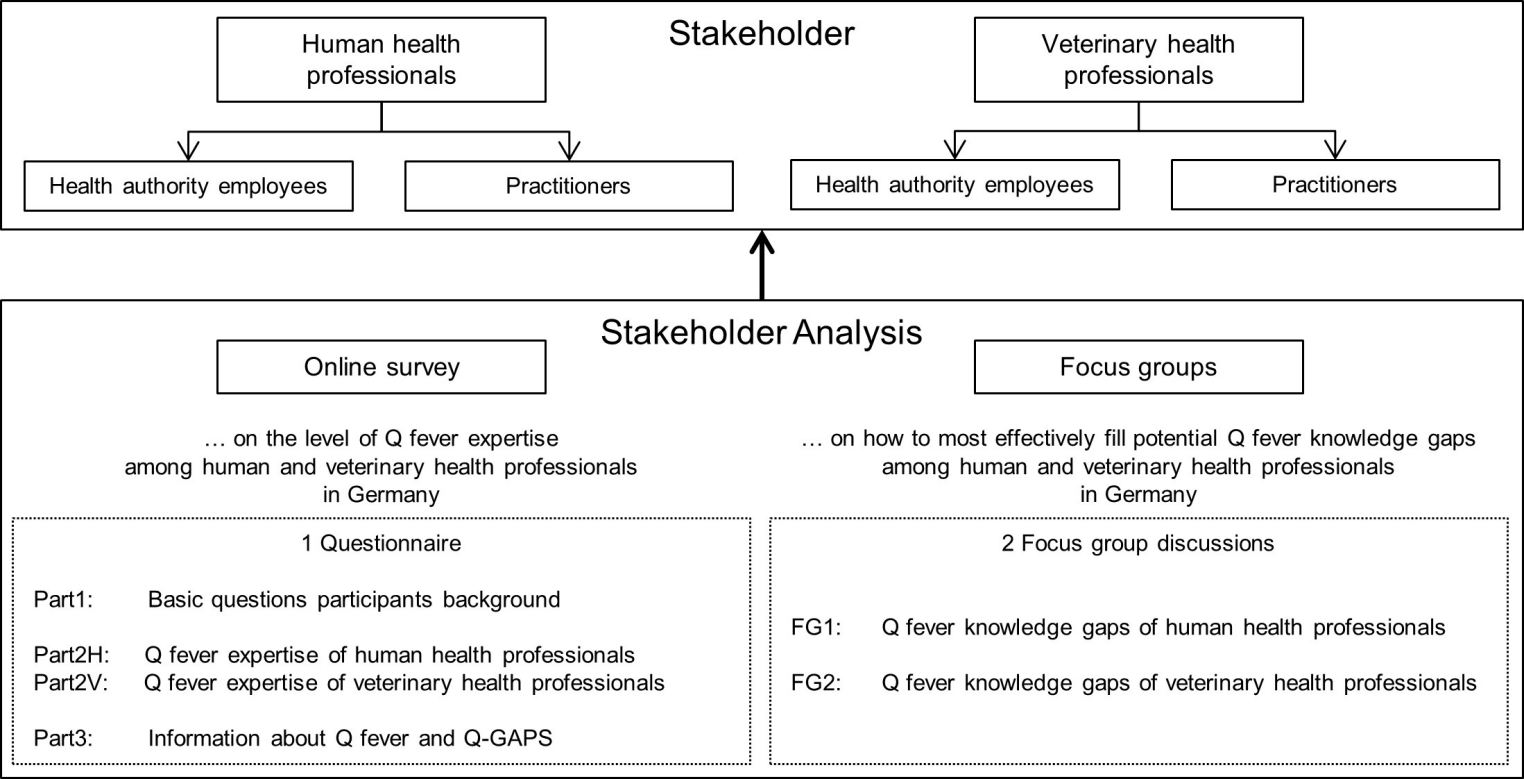
Methodology of this stakeholder analysis.

For this stakeholder analysis, we performed an online survey and two focus group analyses as a combination of methods in qualitative research [29, 30]. To assess knowledge differences and to consider different perspectives and experiences regarding Q fever between stakeholder groups, we investigated the two stakeholder groups “human health professionals” and “veterinary health professionals”, while each of this group was further subdivided into the two groups “practitioners” and “health authority employees” [30, 31]. Moreover, as sporadic human Q fever cases are reported nationwide in Germany, our stakeholder analysis was conducted at the national level. Due to the fact that most Q fever cases in humans and animals were reported within southwestern federal states of Germany [25], we assumed that the extent of knowledge differed between stakeholders who are working in southwestern vs. northeastern parts of Germany. Accordingly, we compared these regions in regard to the online survey results (Fig 1).

#### Online survey

The nationwide, voluntary online survey was addressed to the four previously mentioned stakeholder groups. The survey was conducted from December 15, 2018 until March 15, 2019. We used LimeSurvey® software as a survey tool (LimeSurvey GmbH [2017]/LimeSurvey: An Open Source survey tool/LimeSurvey GmbH, Hamburg, Germany. URL http://www.limesurvey.org). The invitation to our survey included a brief description about the project and a Uniform Resource Locator (URL) link to the survey. To prevent information bias, we did not mention words such as Q fever, coxiellosis, *C*. *burnetii* or small ruminants [32, 33]. Different invitation methods to the survey were used to reach as many stakeholders as possible. We contacted health professionals at the regional health authorities and specialist associations in both the human and veterinary health sectors. Distribution was digitally conducted via e-mail, newsletters and homepages, among other resources; additionally, distribution was analogously conducted via personal contact in an exhibition, as well as through the use of flyers and print media. Participation was voluntary and anonymous and depended on the acceptance of the privacy policy.

The survey questionnaire was split into three parts. Part 1 began with basic questions about the participants’ professional backgrounds. Depending on the participants’ indications in part 1, participants received part 2 of the questionnaire for human health professionals (part 2H) or for veterinary health professionals (part 2V). In this situation, a typical case was used as an example that was tailored to the professional reality of the stakeholder groups. It was followed by questions that were related to the knowledge concerning the diagnosis, control and prevention of Q fever and its zoonotic potential among the participants. We used a validated questionnaire with open and closed questions using a five-point verbal rating scale. Answering the questionnaire was not mandatory to proceed. After finishing part 1 and part 2 of the questionnaire, the participants obtained information about Q fever and the Q fever GermAn Interdisciplinary Program for reSearch (Q-GAPS), under which this study was conducted, via URL links (part 3). The participants were able to leave their e-mail addresses to get informed about further Q-GAPS projects. These voluntary contact details were not connected to the survey answers of the participants (see S1 File).

For the descriptive survey analysis, the composition of the study population and the responses of the participants were determined in terms of absolute numbers and percentages by using SAS software (Version 9.4 of the SAS System for Windows®, Copyright © 2002–2012 SAS Institute Inc. SAS and all other SAS Institute Inc. product or service names are registered trademarks or trademarks of SAS Institute Inc., Cary, NC, USA). Moreover, multivariable logistic regression models were calculated for the influence of the stakeholder group (practitioners vs. health authority employees) and the influence of the region (northeastern Germany vs. southwestern Germany) on the responses of the participating human health professionals and veterinary health professionals, respectively, (PROC LOGISTIC of the SAS System for Windows®, Copyright © 2002–2012 SAS Institute Inc. SAS and all other SAS Institute Inc. product or service names are registered trademarks or trademarks of SAS Institute Inc., Cary, NC, USA).

#### Focus group discussions

Both focus group discussions were conducted at the University for Veterinary Medicine Hanover Foundation (TiHo Hannover) in Hanover, Germany, in May 2019. Due to the assumption of the differences between human and veterinary health professionals regarding their knowledge gaps of Q fever, the focus groups were split for veterinary health professionals and human health professionals.

The participants consisted of practitioners or health authority employees who worked in different regions of Germany. The invitation was issued via the same channels that were used for the survey to reach as many stakeholders as possible. Additionally, participants who quoted their contact details at the end of the online survey were invited. Subsequently, the stakeholders had to register for workshop participation and had to answer basic questions about their professional background (human health professionals vs. veterinary health professionals; practitioners vs. health authority employees; field of specialization; region). As this invitation process produced a low response rate for human health professionals, we then directly contacted certain participants via phone call. We composed the groups heterogeneously (practitioners vs. health authority employees; northeastern Germany vs. southwestern Germany). In total, nine stakeholders participated in each focus group.

Both focus groups were conducted by the first and last authors as moderator and co-moderator, respectively, and were structured by the following same four guiding questions:

1. Which information sources do I generally prefer to acquire knowledge?
2. Which information sources do colleagues in my *>>stakeholder group<<* prefer to acquire knowledge?
3. What is the best way to reach my colleagues in regard to a topic to which they are not sensitized?
4. Which information sources should we use best to close knowledge gaps about Q fever among my >>*stakeholder group*<<?

Participants in each focus group answered the four guiding questions in changing working groups (with two to five participants per working group) while also documenting their answers on presentation cards and pin boards. Subsequently, each working group presented their findings to the fellow participants and discussed them for complementation. The notes that were taken during the focus groups by the co-moderator and the photos of the pin boards were used to document the results of the focus groups. No tape recording was used for documentation [29–31, 34, 35]. For the evaluation of the two focus groups, the basic steps of a qualitative content analysis were conducted. Based on the research question "How can knowledge gaps best be resolved?", we transcribed the photodocumented results of the focus groups into text and conducted an initiating text work. Afterwards, a hierarchical code system was set by using deductive-inductive category development, and the text material was coded. The following analysis was oriented on a case-based thematic summary, and the results were visualized as a theme matrix [36, 37].

### Further quality assurance

To assess the study questions, we followed international guidelines on qualitative research via the performance of stakeholder analyses and focus groups, and we used the COREQ checklist as a guideline for reporting the data [29–31, 34–37]. We referred to the literature that was accessed in a nonsystematic search strategy by using Web of Science (http://apps.webofknowledge.com) as the search database. Publications in the English and German languages were accessed.

## Results

### Composition of the study population

**Fig 2:**
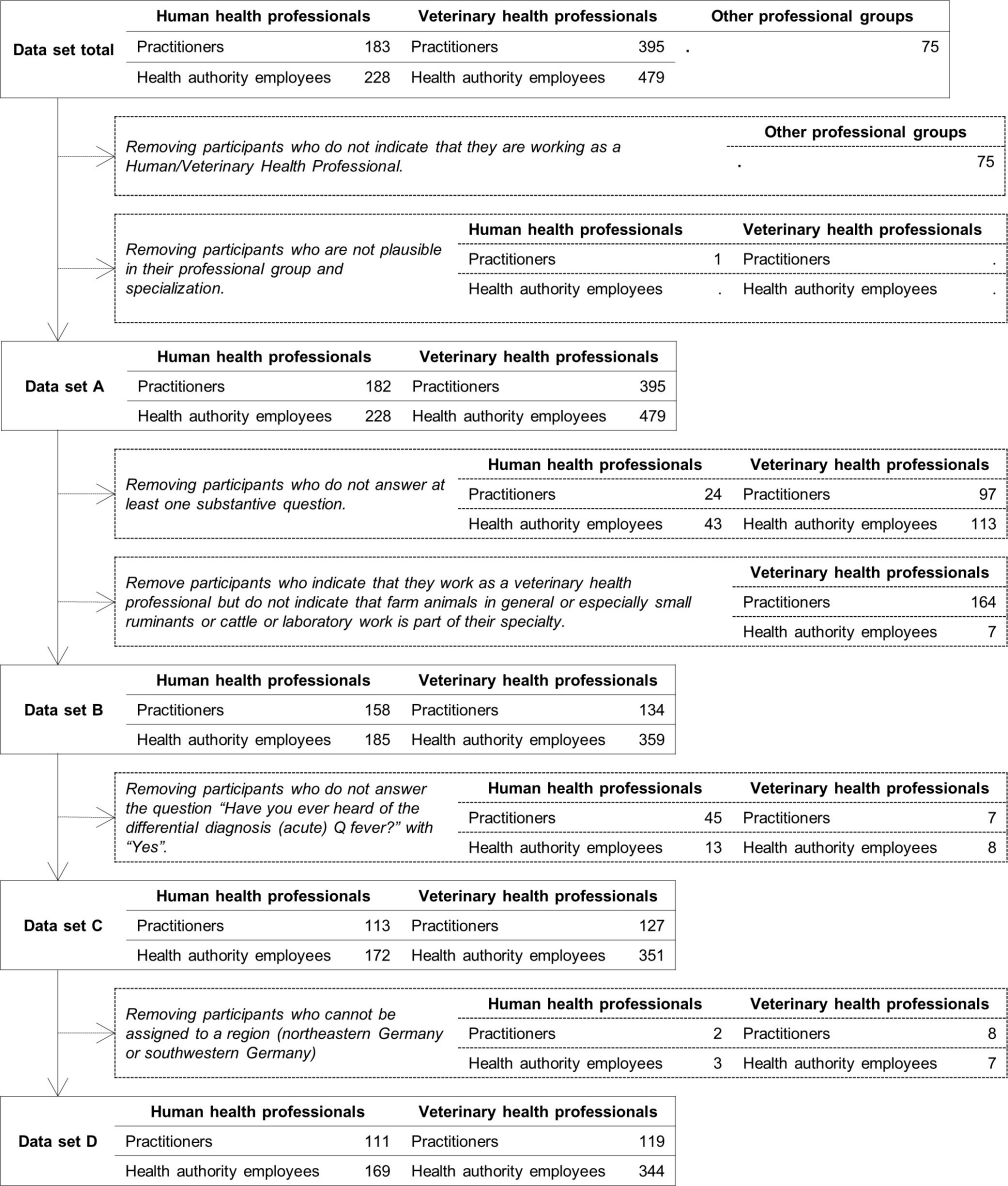
Plausibility check and data sets (online survey).

In total, the convenience sample of the **online survey** included 1,360 participants. After a plausibility check, which included the removal of incongruous participants and unfilled questionnaires, 836 data sets were able to be transferred for further analyses (Fig 2).

Human health practitioners primarily included gynecologists and obstetricians (38.61%, n = 61) followed by general practitioners (24.68%, n = 39) and internists (16.46%, n = 26). Health authority employees were specialized in infection control, hygiene and environmental medicine (24.86%, n = 46), as well as in public health (23.24%, n = 43). Veterinary health practitioners primarily included specialization in farm animals in general (54.48%, n = 73) followed by specialization in cattle specifically (28.36%, n = 38). Only 14.18% (n = 19) of the participants specialized in small ruminants. In comparison, veterinary health authority employees specialized in farm animals in general (80.22%, n = 288) or in the laboratory sector (13.37%; n = 48). Only 4.74% (n = 17) of the employees reliably specialized in small ruminants (see Table 1). Most of the human and veterinary health practitioners worked in northeastern Germany (63.29%; n = 100 and 51.49%; n = 69, respectively), whereas most of the human and veterinary health authority employees worked in southwestern Germany (69.19%; n = 128 and 59.05%; n = 212, respectively; see Table 2).

**Table 1:** Description of human and veterinary health professionals of Germany based on the field of specialization (online survey).

Stakeholder group	Field of specialization the stakeholder work in	Study population	
		n	%
**Human health professionals**			
Practitioners	Gynecology and obstetrics medicine	61	38.61
	General medicine	39	24.68
	Internal medicine	26	16.46
	Other	22	13.92
	Not specified	5	3.16
	Laboratory	4	2.53
	Infection protection, hygiene and environmental medicine	1	0.63
	Total	158	100.00
Health authority employees	Infection protection, hygiene and environmental medicine	46	24.86
	Public health	43	23.24
	Other	30	16.22
	Internal medicine	25	13.51
	Not specified	21	11.35
	General medicine	16	8.65
	Laboratory	3	1.62
	Gynecology and obstetrics medicine	1	0.54
	Total	185	100.00
**Veterinary health professionals**			
Practitioners	Farm animals, in general	73	54.48
	Farm animals, especially cattle	38	28.36
	Farm animals, especially small ruminants	19	14.18
	Laboratory work	4	2.99
	Total	134	100.00
Health authority employees	Farm animals, in general	288	80.22
	Laboratory work	48	13.37
	Farm animals, especially small ruminants	17	4.74
	Farm animals, especially cattle	6	1.67
	Total	359	100.00

n = study population size; Other = other field of specialization than the ones listed here.

**Table 2:** Description of human and veterinary health practitioners of Germany based on the region they work in (online survey).

Stakeholder group	Region the stakeholder work in	Target population		Study population	
		N	%	n	%
**Human health professionals**					
Practitioners	Northeastern Germany^1^	119,898	33.39	100	63.29
	Southwestern Germany^2^	239,201	66.61	55	34.81
	Not specified	-	-	3	1.90
	Total	359,099	100.00	158	100.00
Health authority employees	Northeastern Germany	4,133	41.25	54	29.19
	Southwestern Germany	5,887	58.75	128	69.19
	Not specified	-	-	3	1.62
	Total	10,020	100.00	185	100.00
**Veterinary health professionals**					
Practitioners	Northeastern Germany	7,786	36.70	69	51.49
	Southwestern Germany	13,431	63.30	57	42.54
	Not specified	-	-	8	5.97
	Total	21,217	100.00	134	100.00
Health authority employees	Northeastern Germany	2,822	43.31	140	39.00
	Southwestern Germany	3,694	56.69	212	59.05
	Not specified	-	-	7	1.95
	Total	6,516	100.00	359	100.00

^1^ Berlin, Brandenburg, Bremen, Hamburg, Mecklenburg-West Pomerania, Lower Saxony, Saxony, Saxony-Anhalt, Schleswig-Holstein;

^2^ Baden-Wurttemberg, Bavaria, Hesse, North Rhine-Westphalia, Rhineland-Palatinate, Saarland, Thuringia.

N = target population size; n = study population size

Both focus groups had nine participants each. Although only veterinary health professionals participated in the focus group for veterinary health professionals, the composition of the participants in the focus group for human health professionals was more heterogeneous, as two veterinary health professionals and one epidemiologist participated in this group.

### Basic knowledge of Q fever

When subjected to a typical case report in the online survey, considerably fewer human health professionals than veterinary health professionals considered Q fever as being a differential diagnosis (10.88%, n = 31 vs. 61.09%, n = 292). Although 71.52% (n = 113) of the human health practitioners had already heard of (acute) Q fever as differential diagnoses for the aforementioned symptom complex, this was the case in more than 90% of the human health authority employees and veterinary health professionals (see S2 Table).

According to the logistic regression model, human health authority employees are aware of the term “Q fever” approximately seven times more often than human health practitioners (p = 0.0002; see Table 3).

**Table 3:** Multivariable logistic regression analysis of the proportion of correct answers of human health professionals (online survey).

Question	Variable	Category	Proportion of correct answers/correct and wrong answers (%)	Odds ratio (OR)	95% confidence interval (Cl)	Chi Square p-value	Likelihood-Ratio p-value
**Basic knowledge of human health professionals regarding Q fever**							
Awareness of term Qfever*	Stkh group	HHP	111/143 (77.62)	6.95	2.54–19.01	**0.0002**	**< .0001**
		HHAE	169/174 (97.13)				
	Region	NE	118/147 (80.27)	2.78	1.17–6.60	**0.0205**	
		SW	162/170 (95.29)				
Based on case report: Consideration of Q fever as DD if the participant is aware of term Q fever*	Stkh group	HHP	9/111 (8.11)	1.17	0.49–2.82	0.7225	0.0700
		HHAE	21/169 (12.43)				
	Region	NE	7/118 (5.93)	2.48	0.97–6.31	0.0575	
		SW	23/162 (14.20)				
**Familiarity with risk factors regarding Q fever of human health professionals**							
Human health: Risk estimation for developing acute Q fever if the following events occur							
Visit of markets with animal exhibition*	Stkh group	HHP	54/96 (56.25)	0.96	0.55–1.69	0.8975	0.3667
		HHAE	87/148 (58.78)				
	Region	NE	53/101 (52.48)	1.47	0.84–2.56	0.1740	
		SW	88/143 (61.54)				
Hiking in areas with sheep farming*	Stkh group	HHP	46/92 (50.00)	2.11	1.20–3.74	**0.0097**	**0.0045**
		HHAE	103/147 (70.07)				
	Region	NE	53/97 (54.64)	1.37	0.78–2.42	0.2762	
		SW	96/142 (67.61)				
**Familiarity with diagnosis, control and prevention regarding Q fever of human health professionals**							
Human health: Evaluation of the following statements of acute Q fever							
An illness of acute Q fever during pregnancy can lead to an abortion*	Stkh group	HHP	72/80 (90.00)	2.28	0.737.15	0.1580	0.2407
		HAE	133/139 (95.68)				
	Region	NE	77/84 (91.67)	1.32	0.42–4.10	0.6340	
		SW	128/135 (94.81)				
Outbreaks of acute Q fever in the human population are often associated with diseases of pigeons (wild, city or private pigeon populations)**	Stkh group	HHP	55/75 (73.33)	2.08	0.98–4.40	0.0551	0.0933
		HHAE	119/139 (85.61)				
	Region	NE	67/86 (77.91)	1.11	0.53–2.36	0.7786	
		SW	107/128 (83.59)				
Diagnostic laboratory tests in connection with acute Q fever must be billed privately to the patient**	Stkh group	HHP	89/92 (96.74)	1.68	0.24–11.80	0.6034	0.5554
		HHAE	128/130 (98.46)				
	Region	NE	84/87 (96.55)	1.95	0.29–13.74	0.5007	
		SW	133/135 (98.52)				
Illnesses of acute Q fever should be treated with antibiotics*	Stkh group	HHP	74/86 (86.05)	5.07	1.77–14.50	**0.0025**	**0.0034**
		HHAE	136/143(95.10)				
	Region	NE	88/93 (94.62)	0.26	0.08–0.83	**0.0225**	
		SW	122/136 (89.71)				
According to the IfSG, the clinically diagnosed Q fever should be reported to the responsible health authority before the laboratory result is obtained**	Stkh group	HHP	42/80 (52.50)	2.75	1.46–5.15	**0.0016**	**0.0061**
		HHAE	97/133 (72.93)				
	Region	NE	54/83 (65.06)	0.71	0.38–1.35	0.2985	
		SW	85/130 (65.38)				
According to the IfSG, the responsible health authority will then forward the case of Q fever, which has thus far only been clinically diagnosed, to the competent state authority**	Stkh group	HHP	32/67 (47.76)	2.87	1.49–5.53	**0.0016**	**< .0001**
		HHAE	101/133 (75.94)				
	Region	NE	41/76 (53.95)	1.78	0.93–3.41	0.0815	
		SW	92/124 (74.19)				
Your patient is protected against secondary diseases of acute Q fever by the antibodies detected in the laboratory**	Stkh group	HHP	56/79 (70.89)	0.95	0.48–1.88	0.8912	0.4626
		HHAE	89/122 (72.95)				
	Region	NE	51/76 (67.11)	1.51	0.77–2.97	0.2292	
		SW	94/125 (75.20)				
Acute Q fever can become chronic in some cases and may lead to endocarditis, vasculitis, osteomyelitis, hepatitis, pneumonia or neurological manifestation*	Stkh group	HHP	82/84 (97.62)	0.42	0.08–2.25	0.3140	0.5396
		HHAE	123/130 (94.62)				
	Region	NE	79/82 (96.34)	1.03	0.23–4.56	0.9658	
		SW	126/132 (95.45)				

Significant variables (p < 0.05, likelihood ratio test) are printed in bold.

*Correct answer = Yes, Very high risk/High risk and Agree completely/Rather agree, respectively; wrong answer = No, Low risk/Very low risk and Disagree rather/ Disagree completely, respectively;

**Correct answer = Disagree rather/Disagree completely; wrong answer = Agree completely/Rather agree, respectively;

Stkh group = Stakeholder group;

HHP = Human health practitioners; HHAE = Human health authority employees;

NE = Northeastern Germany (Berlin, Brandenburg, Bremen, Hamburg, Mecklenburg-West Pomerania, Lower Saxony, Saxony, Saxony-Anhalt, Schleswig-Holstein);

SW = Southwestern Germany (Baden-Wurttemberg, Bavaria, Hesse, North Rhine-Westphalia, Rhineland-Palatinate, Saarland, Thuringia);

DD = Differential diagnosis; IfSG = German Protection against Infection Act.

### Familiarity with risk factors regarding Q fever

As a result of the online survey, approximately half of the human health professionals knew that visiting markets with animal exhibitions (50.18%, n = 143) and hiking in areas with sheep farming (52.63%, n = 150) cause very high or high risks for people to become infected with *C*. *burnetii*. Among veterinary health professionals, familiarity with risk factors for the infection of small ruminant flocks varied between the listed risk factors. Two-thirds of the veterinary health professionals correctly considered the exhibition of individual animals of the herd (59.41%, n = 284) as being (very) high risk for a small ruminant flock to become infected with *C*. *burnetii*. In addition, 41.21% (n = 197) of veterinary health professionals correctly estimated the infection risk for a small ruminant flock from grazing on land that was grazed by sheep and goats more than one year ago to be (very) high. Most of the veterinary health professionals correctly recognized that multiple lambing in the same bay, without cleaning and disinfection in between the procedures, may be of (very) high risk for a flock to become infected (81.38%, n = 389; see S3 Table).

According to the logistic regression model, human health authority employees chose the correct answer (very high risk/high risk) regarding the risk of hiking in areas with sheep farming approximately two times more often than human health practitioners (p = 0.0097; see Table 3).

### Familiarity with Q fever diagnosis, control and prevention

In the online survey, most of the human health professionals correctly agreed (completely or rather agreed) with the statement that acute Q fever can become chronic in some cases and may lead to endocarditis, vasculitis, osteomyelitis, hepatitis, pneumonia or neurological manifestation (72.98%; n = 208) and with the statement that illness of acute Q fever should be treated with antibiotics (74.74%; n = 213). The same result applied to the statement that an illness of acute Q fever during pregnancy can lead to an abortion (72.63%, n = 207). Only half of the professionals correctly disagreed (completely or rather disagreed) with the statement that the patient is protected against secondary diseases of acute Q fever by the antibodies detected in the laboratory (51.58%, n = 147; see S4 Table).

In comparison, most of the veterinary health professionals correctly agreed (completely or rather agreed) with the statement that Q fever can proceed subclinically in sheep, despite excretion of the pathogen (80.96%; n = 387). However, only half of them correctly agreed (completely or rather agreed) with the statement that to prevent Q fever losses at the next lambing, the flock should be vaccinated at least three weeks prior to mating (54.39%; n = 260). Most of the veterinary health professionals correctly agreed (completely or rather agreed) with the statement that outbreaks of Q fever in the human population are often associated with events where sheep shearing is demonstrated (61.09%; n = 292). The same result applied to the statement that raw milk products cannot still be marketed after *C*. *burnetii* is directly detectable in 1 of 45 vaginal swab samples within a flock (73.22%; n = 257; see S5 Table).

According to the logistic regression model, human health authority employees chose the correct answer (agree completely/rather agree) regarding the statement “Illness of acute Q fever should be treated with antibiotics.” approximately five times more often than human health practitioners (p = 0025). Moreover, human health authority employees chose the correct answer (disagree completely/rather disagree) regarding the statement “According to the IfSG, the clinically diagnosed Q fever should be reported to the responsible health authority before the laboratory result is obtained.” three times more often than human health practitioners (p = 0.0016). In addition, human health authority employees chose the correct answer (disagree completely/rather disagree) regarding the statement “According to the IfSG, the responsible health authority will then forward the case of Q fever, which has thus far only been clinically diagnosed, to the competent state authority.” approximately three times more often than human health practitioners (p = 0.0016; see Table 3).

### Regional differences

In the case of the online survey, slightly more human health authority employees from southwestern Germany had already heard of acute Q fever as a differential diagnosis than their colleagues from northeastern Germany (96.09%, n = 123 vs. 85.19%, n = 46). In addition, human health professionals from southwestern Germany were slightly more familiar with risk factors for becoming infected with *C*. *burnetii* (visiting markets with animal exhibitions: 54.32%, n = 88 vs. 44.92%, n = 53; hiking in areas with sheep farming: 59.26%, n = 96 vs. 44.92%, n = 53) than their colleagues in northeastern Germany. This result also applied for the correct answers (rather or completely correct) to the questions about diagnosis, control and prevention of Q fever (illness of acute Q fever during pregnancy can lead to an abortion: 79.01%; n = 128 vs. 65.25%; n = 77; your patient is protected against secondary diseases of acute Q fever by the antibodies detected in the laboratory: 58.02%; n = 94 vs. 43.22%; n = 51; acute Q fever is chronic in some cases and can lead to endocarditis, vasculitis, osteomyelitis, hepatitis, pneumonia or neurological manifestation: 77.78%; n = 126 vs. 66.95%; n = 79).

According to the logistic regression model, human health professionals from southwestern Germany know Q fever approximately three times more often than human health professionals from northeastern Germany (p = 0.0205). Moreover, human health authority professionals from southwestern Germany chose the correct answer (agree completely/rather agree) regarding the statement “Illness of acute Q fever should be treated with antibiotics.” only approximately four times less often than human health practitioners from northeastern Germany (p = 0.0225; see Table 3).

Slightly more veterinary health practitioners from northeastern Germany had already heard of Q fever as differential diagnoses than their colleagues from southwestern Germany (97.10%, n = 67 vs. 91.23%, n = 52). More colleagues from northeastern Germany chose the correct statement regarding risk factors for small ruminant flocks becoming infected with *C*. *burnetii* (exhibition of individual animals of the herd: 63.24%; n = 129 vs. 57.92%; n = 150). More colleagues from southwestern Germany chose the correct statements regarding risk factors (grazing on land that was grazed by sheep and goats more than one year ago: 45.56%; n = 118 vs. 36.76%; n = 75; multiple lambing in the same bay, without cleaning and disinfection in between: 85.71%; n = 222 vs. 78.92%; n = 161). Furthermore, veterinary health professionals from southwestern Germany knew the correct answers (rather or completely correct) to the questions about diagnosis, control and prevention of Q fever slightly more often than participants from northeastern Germany (Q fever diseases lead to persistent immunity of the animals after infestation of a herd of small ruminants: 51.35%; n = 133 vs. 43.14%; n = 88; outbreaks of Q fever in the human population are often associated with events where sheep shearing is demonstrated: 64.48%; n = 167 vs. 58.33%; n = 119; to prevent Q fever losses at the next lambing, the flock should be vaccinated at least three weeks prior to mating: 57.53%; n = 149 vs. 52.45%; n = 107).

According to the logistic regression model, veterinary health professionals from southwestern Germany considered of Q fever as differential diagnosis if the participant is aware of term Q fever approximately two times more often than veterinary health professionals from northeastern Germany (0.0255; see Table 4).

**Table 4:** Multivariable logistic regression analysis of the proportion of correct answers of veterinary health professionals (online survey).

Question	Variable	Category	Proportion of correct answers/correct and wrong answers (%)	Odds ratio (OR)	95% confidence interval (Cl)	Chi Square p-value	Likelihood-Ratio p-value
**Basic knowledge regarding Q fever**							
Awareness of term (acute) Q fever*	Stkh group	VHP	119/120 (99.17)	0.93	0.09–9.19	0.9481	0.9701
		VHAE	344/347 (99.14)				
	Region	NE	204/206 (99.03)	1.28	0.18–9.35	0.8068	
		SW	259/261 (99.23)				
Based on case report: Consideration of Q fever as DD* if the participant is aware of term (acute) Q fever*	Stkh group	VHP	64/119 (53.78)	1.46	0.95–2.24	0.0826	**0.0097**
		VHAE	222/344 (64.53)				
	Region	NE	113/204 (55.39)	1.55	1.06–2.27	**0.0255**	
		SW	173/259 (66.80)				
**Familiarity with risk factors regarding Q fever**							
Small ruminants flock health: Risk estimation for developing Q fever if the following events occur							
Exhibition of individual animals of the herd (e.g., breeding shows, animal auctions)*	Stkh group	VHP	74/99 (74.75)	0.85	0.50–1.44	0.5444	0.0842
		VHAE	205/293 (69.97)				
	Region	NE	129/168 (76.79)	0.63	0.40–0.99	0,045	
		SW	150/224 (66.96)				
Grazing on land that was grazed by sheep and goats more than one year ago*	Stkh group	VHP	42/90 (46.67)	1.29	0.79–2.08	0,308	0.1927
		VHAE	151/279 (54.12)				
	Region	NE	75/157 (47.78)	1.33	0.88–2.02	0,183	
		SW	118/212 (55.66)				
Multiple lambing in the same bay, without cleaning and disinfection in between*	Stkh group	VHP	95/101 (94.06)	1.27	0.46–3.54	0,646	0.1090
		VHAE	288/300 (96.00)				
	Region	NE	161/173 (93.06)	2.66	0.97–7.33	0,059	
		SW	222/228 (97.37)				
**Familiarity with diagnosis, control and prevention regarding Q fever**							
Small ruminants flock health: Evaluation of the following statements of Q fever							
Q fever can proceed subclinical in sheep, despite excretion of the pathogen**	Stkh group	VHP	93/95 (97.89)	1.03	0.192–5.46	0,977	0.3273
		VHAE	284/289 (98.27)				
	Region	NE	163/168 (97.02)	3.27	0.62–17.30	0,163	
		SW	214/216 (99.07)				
Q fever diseases lead to persistent immunity of the animals after infestation of a herd of small ruminants**	Stkh group	VHP	50/82 (60.98)	1.17	0.69–1.96	0,564	0.3943
		VHAE	171/261 (65.51)				
	Region	NE	88/145 (60.69)	1.30	0.83–2.04	0,253	
		SW	133/198 (67.17)				
Outbreaks of Q fever in the human population are often associated with events where sheep shearing is demonstrated*	Stkh group	VHP	59/83 (71.08)	1.76	1.0–3.11	0,052	0.0524
		VHAE	227/277 (100.00)				
	Region	NE	119/157 (75.80)	1.39	0.83–2.34	0,215	
		SW	167/203 (82.27)				
Leading symptoms of Q fever in the human population are exanthema, roseoles, papules, blisters and crusts**	Stkh group	VHP	75/81 (92.59)	0.55	0.22–1.35	0,191	0.3830
		VHAE	234/268 (87.31)				
	Region	NE	129/146(88.36)	1,09	0.56–2.12	0,811	
		SW	180/203 (88.67)				
According to TierGesG, the indirect pathogen detection should be reported to the responsible veterinary office**	Stkh group	VHP	34/76 (44.74)	0.99	0.58–1.68	0,971	0.9603
		VHAE	100/226 (44.25)				
	Region	NE	58/126 (46.03)	0.94	0.59–1.49	0,783	
		SW	76/174 (43.68)				
Since *Coxiella burnetii* was detected in only 1 of 45 vaginal swab specimens, Q fever can be excluded as the cause of the flock symptoms**	Stkh group	VHP	83/87 (95.40)	0.90	0.28–2.84	0,852	0.9215
		VHAE	253/267 (94.76)				
	Region	NE	145/152 (95.39)	0.85	0.32–2.27	0,747	
		SW	191/202 (94.55)				
To prevent Q fever losses at the next lambing, the flock should be vaccinated at least 3 weeks prior to covering*	Stkh group	VHP	66/75 (88.00)	0.69	0.32–1.52	0,356	0.6235
		VHAE	190/227 (83.70)				
	Region	NE	107/127 (84.25)	1.11	0.59–2.10	0,749	
		SW	149/175 (85.14)				
Raw milk products can still be marketed after Coxiella burnetii is directly detectable in 1 of 45 vaginal swab samples**	Stkh group	VHP	87/89 (97.75)	0.41	0.09–1.85	0,244	0.4371
		VHAE	252/266 (94.73)				
	Region	NE	148/155 (95.48)	1.10	0.40–3.05	0,855	
		SW	191/200 (95.50)				

Significant variables (p < 0.05, likelihood ratio test) are printed in bold.

*Correct answer = Yes, Very high risk/High risk and Agree completely/Rather agree, respectively; wrong answer = No, Low risk/Very low risk and Disagree rather/Disagree completely, respectively;

**Correct answer = Disagree rather/Disagree completely; wrong answer = Agree completely/Rather agree, respectively;

Stkh group = Stakeholder group;

VHP = Veterinary health practitioners; VHAE = Veterinary health authority employees;

NE = Northeastern Germany (Berlin, Brandenburg, Bremen, Hamburg, Mecklenburg-West Pomerania, Lower Saxony, Saxony, Saxony-Anhalt, Schleswig-Holstein);

SW = Southwestern Germany (Baden-Wurttemberg, Bavaria, Hesse, North Rhine-Westphalia, Rhineland-Palatinate, Saarland, Thuringia);

DD = Differential diagnosis; TierGesG = German National Animal Health Act.

### Closing knowledge gaps

The results of the deductive-inductive category development regarding the transcribed outcome from question 4 “Which information sources should we use best to close knowledge gaps about Q fever among my >>*stakeholder group*<<?” (see S6 Table) are summarized as a theme matrix (see Table 5). This theme matrix shows two levels of category development (level 1>level 2) to qualitatively summarize and clearly present the outcome of the focus group discussions. As the outcome was different for the four stakeholder groups, the matrix lists different categories for each stakeholder group.

**Table 5:** Most suitable information sources to close knowledge gaps about Q fever (focus group discussions).

Stakeholder group	Sub categories	
	Detail Level 1	Detail Level 2
HHP	Direct conversation	• Conversation partners • Conversation tools
	Reading material	• Open Access • Specialist literature
	Seminars	• Academic education • Conferences • E-Learning • Lectures
HHAE	Direct conversation	• Conversation partners • Conversation tools
	Reading material	• Legislation • Official guidance documents • Open Access • Specialist literature
	Seminars	• Conferences • Lectures
VHP	Direct conversation	• Conversation partners • Conversation tools
	Reading material	• Official guidance documents • Open Access • Specialist literature
	Seminars	• Advanced education • Conferences • E-Learning • Workshops
VHAE	Direct conversation	• Conversation partners • Conversation tools
	Reading material	• Open Access • Specialist literature
	Seminars	• Advanced education • Conferences • E-Learning • Lectures • Workshops

HHP = Human health practitioners; HHAE = Human health authority employees;

VHP = Veterinary health practitioners; VHAE = Veterinary health authority employees.

In both focus groups, direct conversation was frequently named as being a useful information source to resolve knowledge gaps. The participants considered personal discussions with intra- and interdisciplinary conversation partners as useful. Moreover, the participants reflected about conversation tools as being useful to keeping the stakeholder groups informed like communication via telephone, e-mail and social media, but also team meetings or other events. Furthermore, the formation of an interdisciplinary Q fever working group per county was mentioned several times for initiating good communication with all of the key stakeholder groups in normal times, i.e., before the first occurrence of Q fever, and to use this foundation for rapid and trustful communications in the event of an epidemic.

Reading materials in the form of specialist literature, legislative texts or official guidance documents were most frequently considered by human health authority employees as being useful sources of information to resolve Q fever knowledge gaps. However, participants’ statements indicated that open access to specialist literature is very important for the other stakeholder groups too. In this context, the participants emphasized that a clear overview of the appropriate literature and information is urgently needed to effectively complete existing knowledge gaps. For example, they considered an open access publication that reviews existing empirical Q fever knowledge to be useful. Furthermore, information sheets and an official catalog of measures were requested to be able to quickly refer to the required knowledge in the event of a Q fever case. Access to information via the Internet was also highlighted as being an effective tool. The participants recommended not only the websites of official institutions such as governmental institutions or specialist associations for reading, but also various (free) apps for smartphones or tablet PCs.

Seminars were also considered to be an important source of information for all of the stakeholder groups. In this scenario, the participants emphasized the importance of academic education, with a stronger focus on zoonoses in general and Q fever in particular. Participants recommended mandatory continuing education on the topic of zoonoses for all stakeholders in order to maintain and deepen the acquired knowledge after graduation. In particular, participants also pointed to seminars that take place as part of the trainee programs of human and veterinary health authority employees. In addition to face-to-face events such as conferences, lectures or workshops, the participants highlighted e-learning seminars as being a very useful source of information. In this scenario, both e-learning events with an interactive exchange of participants and e-learning offers for self-study, which can be flexibly performed in terms of time, were mentioned.

To ensure that the required knowledge actually reaches the stakeholder groups via the aforementioned information sources, the influence of official institutions, such as specialist associations and governmental institutions, was repeatedly emphasized. The participants mentioned that these institutions are responsible for passing on knowledge to the stakeholder groups and for indicating the importance of Q fever and zoonoses in general.

## Discussion

With this stakeholder analysis, we identified what degree of Q fever expertise and the knowledge gaps that human and veterinary health professionals in Germany possess, as well as how these gaps can best be resolved. However, when evaluating the results, we have to consider possible limitations and to discuss the validity and reliability of this stakeholder analysis [30].

In the case of the online survey, the participants were acquired via numerous and different channels, which resulted in a convenience sample; thus, this sample was valid for the participating human and veterinary health professionals solely at the time of the stakeholder analysis. Nevertheless, the size of the study population per each stakeholder group was sufficient for an initial informative impression of Q fever knowledge. We were also initially concerned by the heterogeneity of the focus group for human health professionals. However, while conducting this focus group, we found that the discussions benefited from the interdisciplinary exchange [see Table 2; 32, 33, 38, 39]. Moreover, selection bias must be discussed, as the participants may have been selected due to their interest in the topic of zoonosis. Therefore, it cannot be predicted that the percentage of well-informed participants overestimated the situation in the target population [32, 33, 40]. What was particularly striking was the high degree of willingness to participate among veterinary health professionals. In this scenario, the considerable interest of veterinary health authority employees can be explained by the fact that this stakeholder group is most frequently involved in zoonosis control, as well as in its prevention; therefore, this group was notably sensitized to the survey call. Moreover, the number of participating veterinary health practitioners was comparable. With regard to this stakeholder group, it must be emphasized that the proportion of specialists in small ruminants is limited in Germany; additionally, with 19 participants, an unmeasurable but large proportion of these colleagues participated in the survey. Difficulties in the willingness to participate among human health professionals have to be noted, which could have partially caused nonresponse bias [32, 33]. A common argument of the representatives whom we asked for survey forwarding was that zoonotic diseases are a niche topic in human medicine and that their colleagues are so considerably involved in other topics, such as cardiovascular disease, diabetes or cancer, that this survey would be an unnecessary burden; therefore, the survey could not be distributed. Potential participants also requested an official call from national and federal human health authorities, respectively, to participate in the survey. Therefore, the disparity in the survey support from association boards can be observed in the distribution of the survey participants, in terms of the fields of specialization or the regions that the participants were working in. In comparison, the fact that the institution (TiHo Hannover) from which this survey was distributed is located in northeastern Germany (which may have indicated that the institution had more influence to stakeholders within this region) may have resulted in a high percentage of participation from this part of Germany. Similar problems regarding selection bias resulted with the acquisition of participants for the focus groups [32, 33]. As a reason for refusing a focus group invitation, many human health professionals stated that a workshop on the topic of Q fever was too specialized to schedule. In addition, they lacked the incentive to participate in the form of an officially recognized training certificate. Nevertheless, the evaluation of 836 survey data sets, with more than 100 participants per stakeholder group, as well as two heterogeneous focus groups, may be considered suitable for obtaining a good first impression of the existing expertise and knowledge, along with developing fitted technologies for the transfer of knowledge.

In addition, the composition of the analyst team should be mentioned, as both analysts had to balance their roles as ’insiders’ and ’outsiders’ [30].This conflict was mitigated by the fact that both analysts were researchers and were not practitioners or health authority employees.

In the descriptive analysis, a high proportion of the participants in all of the stakeholder groups had already heard of the term “Q fever”. The differential diagnosis of Q fever was rarely mentioned in the context of the case examples, which was especially noticeable for human health practitioners. Moreover, this observation may be explained by the nonspecific symptoms of Q fever, which may be associated with a variety of differential diagnoses [2, 6]. Conversely, this result also indicates that Q fever is not high on the list of differential diagnoses, based on these symptoms. This scenario may lead to an underreporting of Q fever cases in Germany [25–28]. This scenario also indicates that the awareness of this zoonosis can be enhanced among stakeholder groups by making useful information about Q fever more accessible to them.

The fact that half of the human health professionals could not correctly name the risk factors for Q fever infection correctly and that veterinary health professionals showed a variable knowledge of potential risk factors for small ruminant flocks may impair proper outbreak management and hinder the correct diagnosis, control and prevention of Q fever [41, 42].

With regard to detecting future Q fever cases, it is encouraging that 60–80% of the human health professionals correctly answered questions regarding the diagnosis, control and prevention of Q fever. However, this result has to be discussed in light of the lack of awareness of the disease, as well as the deficient consideration of this disease, given the typical, unspecific symptom complex. As soon as awareness is increased, it is to be expected that existing knowledge concerning the diagnosis, control and prevention of Q fever will be (re)activated. Nevertheless, the zoonotic aspect of Q fever should be emphasized, as only half of the veterinary health practitioners correctly assessed the zoonotic potential of an event where sheep shearing was demonstrated. Veterinary health professionals are an important aspect of zoonotic disease prevention, as they act at the interface of animals, animal owners and consumers. Thus, the communication and development of effective preventive measures in small ruminants for the protection of humans will only be possible if veterinary health practitioners recognize the zoonotic potential of Q fever [42, 43].

In the multivariable logistic regression models, it became obvious that human health authority employees seem to be more aware and more informed of Q fever than human health practitioners are as they were more likely to give correct answers. This could be related to the fact that human health authority employees are regularly involved in the monitoring and surveillance of diseases and therefore come into contact with the issue of Q fever more frequently.

In the descriptive analysis, human health professionals and veterinary health professionals from southwestern Germany were slightly more familiar with risk factors and correctly answered the questions on diagnosis, prevention and control more often than their colleagues from northeastern Germany. Although the differences were not particularly substantial, they can be explained by the fact that Q fever outbreaks have been more frequent in southwestern Germany in the past; therefore, participants can accumulate greater knowledge [23–25]. However, in the logistic regression models no clear effect of the region could be found and Q fever outbreaks can occur unpredictably, as was observed in the Netherlands in 2007 [44], more awareness should be created in both regions of Germany.

As described above, the descriptive analysis and the logistic regression analysis revealed differences between the stakeholder groups and the regions in terms of Q fever knowledge. However, no clear significant relationship between the Q fever knowledge and the stakeholder groups and the two regions, respectively, were found. Thus, the hypothesis that the Q fever knowledge of practitioners vs. health authority employees is different was not confirmed. Additionally, the hypothesis that Q fever knowledge is more pronounced among human health professionals and veterinary health professionals, respectively, in southwestern Germany was not confirmed either. However, our study population is not representative for the distribution of German human health professionals and veterinary health professionals, respectively (divided by stakeholder group and region). Thus, this relationship is also not represented in the logistic regression model.

To close Q fever knowledge gaps, direct conversation at the eye level can be important for the successful control and prevention of Q fever, as well as to effectively and quickly resolve knowledge gaps [45, 46]. This scenario becomes especially important in regard to making decisions in the event of an outbreak. As an example of necessary interdisciplinary information exchange, it should be emphasized that human health authority employees can order measures for the animal sector by means of the IfSG to protect the human population. Conversely, interdisciplinary communication with veterinary health professionals who are experts in small ruminants and who have a good connection to the animal owners is mandatory to order effective measures.

Reading material was mentioned as being a very important source of information, especially for human health authority employees, as this group uses official instructions, such as legislative texts or governmental enactments, as basic materials for their daily working decisions; therefore, this group is accustomed to using reading materials to resolve their knowledge gaps. However, the overall considerable importance of open access publications for all of the stakeholder groups must be emphasized. Prices for specialist journals should not be a barrier to information that have to be transmitted to stakeholder groups. Therefore, researchers are encouraged to publish their results in open access journals so that the latest findings can be read and applied in the field. Furthermore, it must be taken into account that new technologies, such as (medical) apps, are gaining popularity among stakeholder groups for information retrieval. Thus, to ensure the supply of high-quality information on the Internet, such technologies must also be offered by researchers and official institutions.

Seminars as a source of information during academic training are important for creating a knowledge base. In addition, an emphasis should be placed on interdisciplinary collaboration during studies. Although the curricula for human and veterinary medicine have parallels and similar topics, such as zoonoses, food safety and antimicrobial resistance, that affect both subjects, collaborative learning has not yet occurred in Germany. In this case, universities and politics are challenged to offer interdisciplinary seminars for students to create awareness among human and veterinary health professionals for the competencies of the opposite subject and to resolve knowledge gaps during this process. The same recommendation applies to advanced trainings in the form of conferences, lectures or workshops, which are rarely organized on an interdisciplinary basis in Germany. Due to the fact that Q fever is a rather rare disease, as well as the fact that interest in further education, especially among human health professionals, is focused on other diseases, efforts should be made to attract established seminars that are willing to provide information on zoonoses in addition to their special topics. In addition to face-to-face events, e-learning is a sensible alternative for successfully acquiring Q fever knowledge without investing considerable time, money or a business trip. This recommendation is especially advantageous for reaching human and veterinary health practitioners who may be self-employed and are subsequently dependent on flexible training times.

As the implementation of these results is the next important step, the Q-GAPS project has already begun to complete the identified gaps. Therefore, existing structures, such as the “Seminar Veterinary Public Health” at TiHo Hannover, have already been used to promote an interdisciplinary exchange between human and veterinary health professionals by using a combination of lectures and focus groups in February 2020 [47]. Moreover, open access to specialist literature (e.g., publications, guidelines, flyers, etc.) is available via the Q-GAPS homepage (www.q-gaps.de) as an information source. Therefore, the requested review about Q fever in German small ruminants has been published by Q-GAPS members [25]. Moreover, a Q fever guideline that includes the existing empirical knowledge is in the process of being written. Soon, interdisciplinary events will be offered to promote conversations between human and veterinary health professionals. Overall, the results of this stakeholder analysis are already being implemented to resolve knowledge gaps about Q fever among human and veterinary health professionals in the sense of the One Health approach.

## Conclusion

This stakeholder analysis, in the form of an online survey and two focus groups, identified what degree of Q fever expertise human and veterinary health professionals in Germany possess, as well as how potential knowledge gaps can be most effectively resolved.

We have demonstrated that Q fever knowledge already exists but still needs to be increased among members of human and veterinary health professionals. Due to a lack of awareness of this zoonosis, especially among human health practitioners, stakeholders who have heard about Q fever still lack the necessary cross-species knowledge to successfully diagnose, control and prevent this zoonosis. Moreover, differences exist between stakeholder groups regarding their work content that focus on diagnosis vs. prevention and control, as well as the region in which they work. In this scenario, stakeholders in southwestern Germany have slightly better Q fever knowledge than their colleagues in northeastern Germany.

Furthermore, this stakeholder analysis clarified that information needs to be provided in a manner and to an optimal extent, which will enable the successful transfer of knowledge. Information sources to resolve knowledge gaps involve direct conversations between the stakeholder groups, as well as reading materials and seminars. Each of these information sources should focus on interdisciplinary programs to strengthen the cooperation between human and veterinary health professionals and to raise awareness of the strengths of each stakeholder group.

The Q-GAPS project has already implemented the results of this stakeholder analysis, which can increase the awareness of Q fever and resolve knowledge gaps among human and veterinary health professionals.

## Supporting information

S1 File Questionnaire (online survey). (DOCX)

S1 Table Raw data (online survey). (XLSX)

S2 Table Basic knowledge of stakeholder groups regarding Q fever (online survey). N/A = Not answered/Don’t know; *DD = Differential diagnosis.
(DOCX)

S3 Table Familiarity with risk factors regarding Q fever (online survey). HHP = Human health practitioners; HHAE = Human health authority employees; VHP = Veterinary health practitioners; VHAE = Veterinary health authority employees; N/A = Not answered/Don’t know; * = Correct answers.
(DOCX)

S4 Table Familiarity with diagnosis, control and prevention regarding Q fever of Human health professionals (online survey). HHP = Human health practitioners; HHAE = Human health authority employees; IfSG = German Protection against Infection Act; N/A = Not answered/Don’t know; * = Correct answers.
(DOCX)

S5 Table Familiarity with diagnosis, control and prevention regarding Q fever of veterinary health professionals (online survey). VHP = Veterinary health practitioners; VHAE = Veterinary health authority employees; TierGesG = German National Animal Health Act; N/A = Not answered/Don’t know; * = Correct answers.
(DOCX)

S6 Table Category development (focus group discussions). (XLSX)
