# Supplementary material for: Q fever expertise among human and veterinary health professionals in Germany – A stakeholder analysis of knowledge gaps
Source: PLoS One. 2022 Mar 3;17(3):e0264629. doi: 10.1371/journal.pone.0264629 (PMC8893703; doi:10.1371/journal.pone.0264629)
Supplement: S1 File — (DOCX) [file pone.0264629.s001.docx]

**Questionnaire (online survey)**

Part 1 for all participants: Basic questions about the participants' professional background

1.1a: Which professional stakeholder group do you belong?

Human health professionals/practitioners

Human health professionals/health authority employees

Veterinary health professionals/practitioners

Veterinary health professionals/health authority employees

Other professional groups/health authority employees

1.2a: Please, name the field(s) of specialization you work in?

________________________________ (Free text)

Don´t know

1.3a: In which federal state are you active?

Baden-Württemberg

Bavaria

Berlin

Brandenburg

Bremen

Hamburg

Hesse

Mecklenburg-Western Pomerania

Lower Saxony

North Rhine-Westphalia

Rhineland-Palatinate

Saarland

Saxony

Saxony-Anhalt

Schleswig-Holstein

Thuringia

Part 2H for participants who belong to the stakeholder group of human health professionals: Typical example and questions focused on Q fever as a zoonosis

Information to the participants

In the following, we present a case report. We ask you to read it and answer the questions below.

Case report – initial presentation to you as the human health professional:

Your patient is a 52-year-old male. His vaccination status is up to date as recommended by STIKO*. Two years ago, your patient had endocarditis from which he has recovered well. Last year he did not have a stay abroad.

Today your patient presents with the following symptoms: For two days, he has had fever, sweating, nausea and vomiting, extreme exhaustion and headaches that do not subside after taking an analgesic. No other family member shows similar symptoms so far.

*German Permanent Vaccination Commission

2H.1a: Which infectious diseases are the first ones that come to your mind when you read this preliminary report? Please name a maximum of five infectious diseases.

Don´t know

________________________________ (Free text)

Information to the participants

A possible differential diagnosis for the case report just described above is acute Q fever. Q fever is caused by the bacterium "*Coxiella burnetii*".

2H.2a: Have you ever heard of the differential diagnosis acute Q fever?

Don´t know

Yes

No

Information to the participants

We ask you to consider the differential diagnosis acute Q fever for the case report just described.

2H.3a-g: How high do you estimate the risk of a person developing acute Q fever, if the following events occur?

|  |  | Don´t know | Very high risk | Serious risk | Low risk | No risk |
| --- | --- | --- | --- | --- | --- | --- |
| a | Consumption of eggs from organic production. |  |  |  |  | * |
| b | Visit of markets with animal exhibition. |  | * |  |  |  |
| c | Insect bite in southern German regions. |  |  |  |  | * |
| d | Hiking in areas with sheep farming. |  | * |  |  |  |
| e | Participation in events with many people. |  |  |  |  | * |
| f | Contact with people from countries of migration. |  |  |  |  | * |
| g | In-patient stay in hospitals. |  |  |  |  | * |

* = correct answer

Information to the participants

We ask you to consider the differential diagnosis acute Q fever for the case report just described.

2H.4a-d: Please evaluate the following statements based on your assessment of diseases of acute Q fever.

|  |  | Don´t know | Agree completely | Rather agree | Disagree rather | Disagree |
| --- | --- | --- | --- | --- | --- | --- |
| a | An illness of acute Q fever during pregnancy can lead to an abortion. |  | * |  |  |  |
| b | Outbreaks of acute Q fever in the human population are often associated with diseases of pigeons (wild, city or private pigeon populations). |  |  |  |  | * |
| c | Diagnostic laboratory tests in connection with acute Q fever must be billed privately to the patient. |  |  |  |  | * |
| d | Illnesses of acute Q fever should be treated with antibiotics. |  | * |  |  |  |

* = correct answer

Information to the participants

The known case report is still valid, but the following questions refer to the period 3 weeks after the first presentation.

Case report – three weeks after initial presentation:

In the last three weeks since your patient's initial presentation, the described symptoms have subsided in a self-limiting manner.

You still want to check your differential diagnosis of acute Q fever and have a serum sample from your patient tested for antibodies.

The laboratory result is "Specific antibodies against *Coxiella burnetii* detectable."

2H.5a-d: How do you rate the following statements regarding the laboratory result above?

|  |  | Don´t know | Agree completely | Rather agree | Disagree rather | Disagree |
| --- | --- | --- | --- | --- | --- | --- |
| a | According to the IfSG, the clinically diagnosed Q fever should be reported to the responsible health authority before the laboratory result is obtained. |  |  |  |  | * |
| b | According to the IfSG, the responsible health authority will then forward the case of Q fever, which has so far only been clinically diagnosed, to the competent state authority. |  |  |  |  | * |
| c | Your patient is protected against secondary or secondary diseases of acute Q fever by the antibodies detected in the laboratory. |  |  |  |  | * |
| d | Acute Q fever can become chronic in some cases and may lead to endocarditis, vasculitis, osteomyelitis, hepatitis, pneumonia or neurological manifestation. |  | * |  |  |  |

* = correct answer; IfSG = German Protection against Infection Act

Part 2V for participants who belong to the stakeholder group of veterinary health professionals: Typical example and questions focused on Q fever as a zoonosis

Information to the participants

In the following, we present a case report. We ask you to read it and answer the questions below.

Case Report – initial presentation to you as the veterinary health professional:

A shepherd contacts you regarding his flock of small ruminants. The flock consists of 200 sheep ewes with their lambs and 50 gimmers as well as 10 goat ewes with their lambs. The flock is kept on changing pastures, which are located near a residential area. Carrying animals are kept for lambing (seasonal from December to April). In the last breeding season, four off-farm, graded sheep rams and one off-farm, graded goat ram were used.

In the current lambing season, the shepherd reports weak-born lambs and placental retention in the sheep and goats. For the goats, the shepherd also reports about four abortions and two stillbirths.

2V.1a: Which infectious diseases are the first ones that come to your mind when you read this preliminary report? Please name a maximum of five infectious diseases.

Don´t know

________________________________ (Free text)

Information to the participants

A possible differential diagnosis for the case report just described is "coxiellosis" or also called Q fever. "Coxiellosis / Q fever" is caused by the bacterium "*Coxiella burnetii*".

2V.2a: Have you ever heard of the differential diagnosis "coxiellosis / Q fever"?

Don´t know

Yes

No

Information to the participants

We ask you to consider the differential diagnosis "coxiellosis / Q fever" for the case report just described.

2V.3a-h: How high do you estimate the risk of a flock of small ruminants developing "coxiellosis / Q fever" if the following events occur?

|  |  | Don´t know | Very high risk | Serious risk | Low risk | No risk |
| --- | --- | --- | --- | --- | --- | --- |
| a | Ingestion of copper-rich mineral feed. |  |  |  |  | * |
| b | Exhibition of individual animals of the herd (e.g. breeding shows, animal auctions). |  | * |  |  |  |
| c | Heavy mosquito infestation before deck season. |  |  |  |  | * |
| d | Grazing on land that was grazed by sheep and goats more than one year ago. |  | * |  |  |  |
| e | Direct contact with people (e.g. petting zoo, markets, farm festivals, ...). |  |  |  |  | * |
| f | Severe endoparasitosis caused by Haemonchus contortus during pregnancy. |  |  |  |  | * |
| g | Walkers with dogs along the pasture. |  |  |  |  | * |
| h | Multiple lambing in the same bay, without cleaning and disinfection in between. |  | * |  |  |  |

* = correct answer

Information to the participants

We ask you to consider the differential diagnosis "coxiellosis / Q fever" for the case report just described.

2V.4a-d: Please evaluate the following statements based on your assessment of diseases of "coxiellosis / Q fever".

|  |  | Don´t know | Agree completely | Rather agree | Disagree rather | Disagree |
| --- | --- | --- | --- | --- | --- | --- |
| a | Q fever can proceed subclinical in sheep, despite excretion of the pathogen. |  | * |  |  |  |
| b | Q fever diseases lead to persistent immunity of the animals after infestation of a herd of small ruminants. |  |  |  |  | * |
| c | Outbreaks of Q fever in the human population are often associated with events where sheep shearing is demonstrated. |  | * |  |  | * |
| d | Leading symptoms of Q fever in the human population are exanthema, roseoles, papules, blisters and crusts]. |  |  |  |  | * |

* = correct answer

Information to the participants

The known case report is still valid, but the following questions refer to the period after the laboratory examination for "coxiellosis / Q fever".

Case report – after laboratory examination for "coxiellosis / Q fever":

You want to check your differential diagnosis of "coxiellosis / Q fever" and have several vaginal swab samples from freshly lambed sheep and goats tested in the laboratory for Q fever pathogens.

The laboratory result is "directly detectable *Coxiella burnetii* in 1 of 45 vaginal swab samples".

2V.5a-d: How do you rate the following statements regarding the laboratory result?

|  |  | Don´t know | Agree completely | Rather agree | Disagree rather | Disagree |
| --- | --- | --- | --- | --- | --- | --- |
| a | According to TierGesG, the indirect pathogen detection should be reported to the responsible veterinary office. |  |  |  |  | * |
| b | Since *Coxiella burnetii* was detected in only 1 of 45 vaginal swab specimens, Q fever can be excluded as the cause of the flock symptoms. |  |  |  |  | * |
| c | In order to prevent Q fever losses at the next lambing, the flock should be vaccinated at least 3 weeks prior to covering. |  | * |  |  |  |
| d | Raw milk products can still be marketed. |  |  |  |  | * |

* = correct answer; TierGesG = German National Animal Health Act

Part 3 for all participants: Questionnaire ending with further information to Q fever and Q‑GAPS

**3.1: Are you interested in participating in a workshop? Would you like to receive more information about the planned workshops? Then please enter your e-mail address* in the field below or send an e-mail to the following address**

________________________________ (Free text)*

Mail to [Fenja.winter@tiho-hannover.de](mailto:Fenja.winter@tiho-hannover.de)

We appreciate your interest and commitment.

*We will not associate your e-mail address with the questions you have just answered and will not share it with any third party. We will be happy to inform you about the planned workshops for human and veterinary health professionals via your e-mail address. Providing your e-mail address is not binding for workshop participation. You may request deletion of your e-mail address from our records at any time. Please feel free to contact [Fenja.winter@tiho-hannover.de](mailto:Fenja.winter@tiho-hannover.de) for this purpose.

**3.2: Thank you for your participation! Would you like to learn more about Q fever?**

Then visit the Q-GAPS homepage [www.q-gaps.de](http://www.q-gaps.de)

What other projects does the BMBF fund on the topic of common diseases/infectious diseases? Visit the BMBF homepage <https://www.gesundheitsforschung-bmbf.de/de/foerderkatalog-2435.php#Volkskra>
