# Supplementary material for: Q fever expertise among human and veterinary health professionals in Germany – A stakeholder analysis of knowledge gaps
Source: PLoS One. 2022 Mar 3;17(3):e0264629. doi: 10.1371/journal.pone.0264629 (PMC8893703; doi:10.1371/journal.pone.0264629)
Supplement: S2 Table — N/A = Not answered/Don’t know; *DD = Differential diagnosis. (DOCX) [file pone.0264629.s003.docx]

**S3 Table. Basic knowledge of stakeholder groups regarding Q fever (online survey).**

| Stakeholder group | Yes | | No | | N/A | | Total | |
| --- | --- | --- | --- | --- | --- | --- | --- | --- |
|  | N | % | N | % | N | % | N | % |
| **Awareness of term (acute) Q fever** | | | | | | | | |
| Human health practitioners | 113 | 71.52 | 33 | 20.89 | 12 | 7.60 | 158 | 100.00 |
| Human health authority employees | 172 | 92.97 | 5 | 2.70 | 8 | 4.32 | 185 | 100.00 |
| Veterinary health practitioners | 127 | 94.78 | 1 | 0.75 | 6 | 4.48 | 134 | 100.00 |
| Veterinary health authority employees | 351 | 97.77 | 3 | 0.84 | 5 | 1.39 | 359 | 100.00 |
| **Based on case report: Consideration of Q fever as DD* if the participant is aware of term (acute) Q fever** | | | | | | | | |
| Human health practitioners | 9 | 7.96 | 104 | 92.04 | . | . | 113 | 100.00 |
| Human health authority employees | 21 | 12.21 | 151 | 87.79 | . | . | 172 | 100.00 |
| Veterinary health practitioners | 65 | 51.18 | 62 | 48.82 | . | . | 127 | 100.00 |
| Veterinary health authority employees | 227 | 64.67 | 124 | 35.33 | . | . | 351 | 100.00 |
