# Supplementary material for: Q fever expertise among human and veterinary health professionals in Germany – A stakeholder analysis of knowledge gaps
Source: PLoS One. 2022 Mar 3;17(3):e0264629. doi: 10.1371/journal.pone.0264629 (PMC8893703; doi:10.1371/journal.pone.0264629)
Supplement: S3 Table — HHP = Human health practitioners; HHAE = Human health authority employees; VHP = Veterinary health practitioners; VHAE = Veterinary health authority employees; N/A = Not answered/Don’t know; * = Correct answers. (DOCX) [file pone.0264629.s004.docx]

**S4 Table. Familiarity with risk factors regarding Q fever (online survey).**

| Stakeholder group | Very high risk | | | | High risk | | | | Low risk | | | | No risk | | | | N/A | | | | Total | | |
| --- | --- | --- | --- | --- | --- | --- | --- | --- | --- | --- | --- | --- | --- | --- | --- | --- | --- | --- | --- | --- | --- | --- | --- |
|  | N | | % | | N | | % | | N | | % | | N | | % | | N | | % | | N | | % |
| **Human health: Risk estimation for developing acute Q fever if the following events occur** | | | | | | | | | | | | | | | | | | | | | | | |
| Visit of markets with animal exhibition | | | | | | | | | | | | | | | | | | | | | | | |
| HHP | *7 | *6.19 | | 48 | | 42.48 | | 38 | | 33.63 | | 4 | | 3.54 | | 16 | | 14.16 | | 113 | | 100.00 | |
| HHAE | *38 | *22.09 | | 50 | | 29.07 | | 59 | | 34.30 | | 2 | | 1.16 | | 23 | | 13.38 | | 172 | | 100.00 | |
| Hiking in areas with sheep farming | | | | | | | | | | | | | | | | | | | | | | | |
| HHP | *15 | *13.27 | | 31 | | 27.43 | | 41 | | 36.28 | | 6 | | 5.31 | | 20 | | 17.70 | | 113 | | 100.00 | |
| HHAE | *50 | *29.07 | | 54 | | 31.40 | | 40 | | 23.26 | | 4 | | 2.33 | | 24 | | 13.95 | | 172 | | 100.00 | |
| **Small ruminants flock health: Risk estimation for developing Q fever if the following events occur** | | | | | | | | | | | | | | | | | | | | | | | |
| Exhibition of individual animals of the herd (e.g., breeding shows, animal auctions) | | | | | | | | | | | | | | | | | | | | | | | |
| VHP | *24 | *18.90 | | 52 | | 40.94 | | 24 | | 18.90 | | 5 | | 3.94 | | 22 | | 17.33 | | 127 | | 100.00 | |
| VHAE | *62 | *17.66 | | 146 | | 41.60 | | 84 | | 23.93 | | 5 | | 1.42 | | 54 | | 15.39 | | 351 | | 100.00 | |
| Grazing on land that was grazed by sheep and goats more than one year ago | | | | | | | | | | | | | | | | | | | | | | | |
| VHP | *14 | *11.02 | | 30 | | 23.62 | | 35 | | 27.56 | | 16 | | 12.60 | | 32 | | 25.20 | | 127 | | 100.00 | |
| VHAE | *41 | *11.68 | | 112 | | 31.91 | | 100 | | 28.49 | | 30 | | 8.55 | | 68 | | 19.37 | | 351 | | 100.00 | |
| Multiple lambing in the same bay, without cleaning and disinfection in between | | | | | | | | | | | | | | | | | | | | | | | |
| VHP | *77 | *60.63 | | 22 | | 17.326 | | 6 | | 4.72 | | 2 | | 1.57 | | 20 | | 15,75 | | 127 | | 100.00 | |
| VHAE | *227 | *64.67 | | 63 | | 17.95 | | 9 | | 2.56 | | 4 | | 1.14 | | 48 | | 13.68 | | 351 | | 100.00 | |
