# Supplementary material for: Q fever expertise among human and veterinary health professionals in Germany – A stakeholder analysis of knowledge gaps
Source: PLoS One. 2022 Mar 3;17(3):e0264629. doi: 10.1371/journal.pone.0264629 (PMC8893703; doi:10.1371/journal.pone.0264629)
Supplement: S4 Table — HHP = Human health practitioners; HHAE = Human health authority employees; IfSG = German Protection against Infection Act; N/A = Not answered/Don’t know; * = Correct answers. (DOCX) [file pone.0264629.s005.docx]

**S5 Table. Familiarity with diagnosis, control and prevention regarding Q fever of Human health professionals (online survey).**

| Stakeholder group | Agree completely | | | | Rather agree | | | | Disagree rather | | | | Disagree completely | | | | N/A | | | | Total | | |
| --- | --- | --- | --- | --- | --- | --- | --- | --- | --- | --- | --- | --- | --- | --- | --- | --- | --- | --- | --- | --- | --- | --- | --- |
|  | N | | % | | N | | % | | N | | % | | N | | % | | N | | % | | N | | % |
| **Human health: Evaluation of the following statements of acute Q fever** | | | | | | | | | | | | | | | | | | | | | | | |
| An illness of acute Q fever during pregnancy can lead to an abortion | | | | | | | | | | | | | | | | | | | | | | | |
| HHP | *39 | *34.51 | | 34 | | 30.09 | | 7 | | 6.19 | | 1 | | 0.88 | | 32 | | 28.32 | | 113 | | 100.00 | |
| HHAE | *95 | *55.23 | | 39 | | 22.67 | | 4 | | 2.33 | | 2 | | 1.16 | | 32 | | 18.61 | | 172 | | 100.00 | |
| Outbreaks of acute Q fever in the human population are often associated with diseases of pigeons (wild, city or private pigeon populations) | | | | | | | | | | | | | | | | | | | | | | | |
| HHP | 1 | 0.88 | | 19 | | 16.81 | | 19 | | 16.81 | | *38 | | *33.63 | | 36 | | 31.58 | | 113 | | 100.00 | |
| HHAE | 6 | 3.49 | | 15 | | 8.72 | | 37 | | 21.51 | | *82 | | *47.67 | | 32 | | 18.60 | | 172 | | 100.00 | |
| Diagnostic laboratory tests in connection with acute Q fever must be billed privately to the patient | | | | | | | | | | | | | | | | | | | | | | | |
| HHP | . | . | | 3 | | 2.65 | | 16 | | 14.16 | | *75 | | *66.37 | | 19 | | 16.81 | | 113 | | 100.00 | |
| HHAE | . | . | | 2 | | 1.16 | | 12 | | 6.98 | | *117 | | *68.02 | | 41 | | 23.83 | | 172 | | 100.00 | |
| Illnesses of acute Q fever should be treated with antibiotics | | | | | | | | | | | | | | | | | | | | | | | |
| HHP | *60 | *53.10 | | 16 | | 14.16 | | 6 | | 5.31 | | 6 | | 5.31 | | 25 | | 22.12 | | 113 | | 100.00 | |
| HHAE | *125 | *72.67 | | 12 | | 6.98 | | 1 | | 0.58 | | 6 | | 3.49 | | 28 | | 16.28 | | 172 | | 100.00 | |
| According to the IfSG, the clinically diagnosed Q fever should be reported to the responsible health authority before the laboratory result is obtained | | | | | | | | | | | | | | | | | | | | | | | |
| HHP | 18 | 15.93 | | 20 | | 17.70 | | 13 | | 11.50 | | *30 | | *26.55 | | 32 | | 28.31 | | 113 | | 100.00 | |
| HHAE | 26 | 15.12 | | 10 | | 5.81 | | 6 | | 3.49 | | *91 | | *52.91 | | 39 | | 22.67 | | 172 | | 100.00 | |
| According to the IfSG, the responsible health authority will then forward the case of Q fever, which has thus far only been clinically diagnosed, to the competent state authority | | | | | | | | | | | | | | | | | | | | | | | |
| HHP | 18 | 15.93 | | 17 | | 15.04 | | 10 | | 8.85 | | *22 | | *19.47 | | 46 | | 40.70 | | 113 | | 100.00 | |
| HHAE | 24 | 13.95 | | 9 | | 5.23 | | 8 | | 4.65 | | *93 | | *54.07 | | 38 | | 22.10 | | 172 | | 100.00 | |
| Your patient is protected against secondary diseases of acute Q fever by the antibodies detected in the laboratory | | | | | | | | | | | | | | | | | | | | | | | |
| HHP | 2 | 1.77 | | 21 | | 18.58 | | 28 | | 24.78 | | *30 | | *26.55 | | 32 | | 28.32 | | 113 | | 100.00 | |
| HHAE | 14 | 8.14 | | 20 | | 11.63 | | 32 | | 18.60 | | *57 | | *33.14 | | 49 | | 28.49 | | 172 | | 100.00 | |
| Acute Q fever can become chronic in some cases and may lead to endocarditis, vasculitis, osteomyelitis, hepatitis, pneumonia or neurological manifestation | | | | | | | | | | | | | | | | | | | | | | | |
| HHP | *52 | *46.02 | | 32 | | 28.32 | | 1 | | 0.88 | | 1 | | 0.88 | | 27 | | 23.89 | | 113 | | 100.00 | |
| HHAE | *95 | *55.23 | | 29 | | 16.86 | | 3 | | 1.74 | | 4 | | 2.33 | | 41 | | 23.83 | | 172 | | 100.00 | |
