# Supplementary material for: Q fever expertise among human and veterinary health professionals in Germany – A stakeholder analysis of knowledge gaps
Source: PLoS One. 2022 Mar 3;17(3):e0264629. doi: 10.1371/journal.pone.0264629 (PMC8893703; doi:10.1371/journal.pone.0264629)
Supplement: S5 Table — VHP = Veterinary health practitioners; VHAE = Veterinary health authority employees; TierGesG = German National Animal Health Act; N/A = Not answered/Don’t know; * = Correct answers. (DOCX) [file pone.0264629.s006.docx]

**S6 Table. Familiarity with diagnosis, control and prevention regarding Q fever of veterinary health professionals (online survey).**

| Stakeholder group | Agree completely | | | | Rather agree | | | | Disagree rather | | | | Disagree completely | | | | N/A | | | | Total | | |
| --- | --- | --- | --- | --- | --- | --- | --- | --- | --- | --- | --- | --- | --- | --- | --- | --- | --- | --- | --- | --- | --- | --- | --- |
|  | N | | % | | N | | % | | N | | % | | N | | % | | N | | % | | N | | % |
| **Small ruminants flock health: Evaluation of the following statements of Q fever** | | | | | | | | | | | | | | | | | | | | | | | |
| Q fever can proceed subclinical in sheep, despite excretion of the pathogen | | | | | | | | | | | | | | | | | | | | | | | |
| VHP | *74 | *58.27 | | 25 | | 19.69 | | 2 | | 1.57 | | . | | . | | 26 | | 20.47 | | 127 | | 100.00 | |
| VHAE | *223 | *63.53 | | 65 | | 18.52 | | 4 | | 1.14 | | 2 | | 0.57 | | 57 | | 16.23 | | 351 | | 100.00 | |
| Q fever diseases lead to persistent immunity of the animals after infestation of a herd of small ruminants | | | | | | | | | | | | | | | | | | | | | | | |
| VHP | 9 | 7.09 | | 26 | | 20.47 | | 35 | | 27.56 | | *18 | | *14.17 | | 39 | | 30.71 | | 127 | | 100.00 | |
| VHAE | 29 | 8.26 | | 63 | | 17.95 | | 98 | | 27.92 | | *75 | | *21.37 | | 87 | | 24.50 | | 351 | | 100.00 | |
| Outbreaks of Q fever in the human population are often associated with events where sheep shearing is demonstrated | | | | | | | | | | | | | | | | | | | | | | | |
| VHP | *28 | *22.05 | | 33 | | 25.98 | | 21 | | 16.54 | | 6 | | 4.72 | | 39 | | 30.71 | | 127 | | 100.00 | |
| VHAE | *119 | *33.90 | | 112 | | 31.91 | | 41 | | 11.68 | | 10 | | 2.85 | | 69 | | 19.66 | | 351 | | 100.00 | |
| Leading symptoms of Q fever in the human population are exanthema, roseoles, papules, blisters and crusts | | | | | | | | | | | | | | | | | | | | | | | |
| VHP | 3 | 2.36 | | 4 | | 3.15 | | 14 | | 11.02 | | *66 | | *51.97 | | 40 | | 31.50 | | 127 | | 100.00 | |
| VHAE | 14 | 3.99 | | 21 | | 5.98 | | 47 | | 13.39 | | *191 | | *54.42 | | 78 | | 22.22 | | 351 | | 100.00 | |
| According to TierGesG, the indirect pathogen detection should be reported to the responsible veterinary office | | | | | | | | | | | | | | | | | | | | | | | |
| VHP | 37 | 29.13 | | 8 | | 6.30 | | 8 | | 6.30 | | *28 | | *22.05 | | 46 | | 36.22 | | 127 | | 100.00 | |
| VHAE | 94 | 26.78 | | 34 | | 9.68 | | 12 | | 3.42 | | *90 | | *25.64 | | 121 | | 34.48 | | 351 | | 100.00 | |
| Since *Coxiella burnetii* was detected in only 1 of 45 vaginal swab specimens, Q fever can be excluded as the cause of the flock symptoms | | | | | | | | | | | | | | | | | | | | | | | |
| VHP | 3 | 2.36 | | 3 | | 2.36 | | 27 | | 21.26 | | *60 | | *47.24 | | 34 | | 26.77 | | 127 | | 100.00 | |
| VHAE | 2 | 0.57 | | 12 | | 3.42 | | 58 | | 16.52 | | *200 | | *56.98 | | 79 | | 22.50 | | 351 | | 100.00 | |
| To prevent Q fever losses at the next lambing, the flock should be vaccinated at least 3 weeks prior to covering | | | | | | | | | | | | | | | | | | | | | | | |
| VHP | *34 | *26.77 | | 34 | | 26.77 | | 4 | | 3.15 | | 7 | | 5.51 | | 48 | | 37.80 | | 127 | | 100.00 | |
| VHAE | *107 | *30.48 | | 85 | | 24.22 | | 18 | | 5.13 | | 20 | | 5.70 | | 121 | | 34.48 | | 351 | | 100.00 | |
| Raw milk products can still be marketed after *Coxiella burnetii* is directly detectable in 1 of 45 vaginal swab samples | | | | | | | | | | | | | | | | | | | | | | | |
| VHP | . | . | | 2 | | 1.57 | | 17 | | 13.39 | | *74 | | *58.27 | | 34 | | 26.77 | | 127 | | 100.00 | |
| VHAE | 4 | 1.14 | | 10 | | 2.85 | | 33 | | 9.40 | | *224 | | *63.82 | | 80 | | 22.79 | | 351 | | 100.00 | |
